# Plasma Free Amino Acids and Risk of Cardiovascular Disease in Chinese Patients With Type 2 Diabetes

**DOI:** 10.3389/fendo.2020.519923

**Published:** 2021-04-14

**Authors:** Tie Li, Hui-Huan Luo, Xiao-Fei Feng, Yu Bai, Zhong-Ze Fang, Gui-Gang Wu, Jian-Lin Wu

**Affiliations:** ^1^ Department of Urology Surgery, Zhongshan Hospital of Dalian University, Dalian, China; ^2^ Department of Toxicology and Sanitary Chemistry, School of Public Health, Tianjin Medical University, Tianjin, China; ^3^ Department of Radiology, Affiliated Zhongshan Hospital of Dalian University, Dalian, China

**Keywords:** cardiovascular diseases, amino acids, metabolism, type 2 diabetes, Chinese

## Abstract

**Objectives:**

This study aimed to explore associations between plasma free amino acids (PFAA) and risk of cardiovascular disease (CVD) in Chinese with Type 2 diabetes (T2D).

**Methods:**

We retrieved 741 inpatients with T2D consecutively from tertiary hospital. Twenty-three PFAA were measured. CVD was defined as having coronary heart disease (CHD) or stroke. Principal component analysis was used to extract factors of PFAA. Factors and their components were introduced into binary logistic regressions as continuous and tertiles to obtain OR (odds ratio) and 95% confidence interval (CI) for CVD (or its components) risk.

**Results:**

Of 741 inpatients, 282 (38.1%) had CVD (CHD alone: 122, stroke alone: 109, both: 51). Five factors were extracted, accounting for 65% of the total variance. Factor 3 composed of glutamate and tryptophan was associated with increased CVD risk (ORs, 95%CI of top vs. bottom tertiles: 1.60, 1.02–2.50 for CVD; 2.19, 1.17–4.07 for stroke, 1.51, 0.83–2.73 for CHD); the ORs (top vs. bottom tertiles) of glutamate were 2.62 (95%CI, 1.18–5.84) for stroke and 1.44 (0.80–2.61) for CHD; the ORs (top vs. bottom tertiles) of tryptophan were 1.50 (0.81–2.75) for stroke and 1.07 (0.58–1.97) for CHD. Comparable results were observed according to important confounders (all P for interaction >0.05).

**Conclusions:**

Elevated factor 3 composed of glutamate and tryptophan was associated with increased CVD, especially stroke in T2D in China.

## Highlights

- This study was conducted in the context of type 2 diabetes (T2D) and provided role of amino acids on cardiovascular diseases (CVD) risk in T2D.
- Data in china was limited. This study provided evidence for CVD prevention of Chinese T2D patients.
- This study found that elevated glutamate and tryptophan were associated with increased CVD, especially stroke in T2D.

## Introduction

Cardiovascular disease (CVD) is one of the most severe complications of type 2 diabetes mellitus (T2D), which accounted for more than 20% all-cause death among patients with T2D in China (1). Researchers had attempted to identify potential modifiable risk factors to improve this situation. In this connection, a prospective study in Hong Kong revealed that optimal control of glycemia, blood pressure, and lipid control could only partially reduce risk of CVD and the residual risk of CVD was still substantial (2). Thus, it is warranted to investigate the possible biological link between T2D and CVD from another angle to generate new insights to help us understand CVD in T2D.

Since the development of metabolomics, we were allowed to explore role of series of metabolites from a novel sight in diseases, including CVD (3). Amino acids participate in protein synthesis, energy metabolism and serve as signaling molecules, which indicate their diverse roles in metabolic homeostasis. For a long time, abnormal amino acids metabolism has been related to vascular function in general population inconsistently, suggesting a subtle and complex relationship between amino acids metabolism and CVD (4, 5). In patients with diabetes, amino acids were markedly changed (6) and combination of amino acids was reported to predict CVDs in T2D (7). However, how amino acids homeostasis influenced vascular and the underlying mechanisms were scarcely investigated. Only a prospective study measured several amino acids in diabetic cohorts and found no associations between these amino acids and macrovascular diseases (8). The relationship of AA with CVD in T2D was largely unknown.

We conducted a hospital-based cross-sectional survey of Chinese inpatients with T2D to explore the global pattern of plasma free amino acids (PFAA) and their associations with risk of CVD and its components, coronary heart disease (CHD) and stroke.

## Materials and Method

### Research Design and Study Patients

The study patients and methods were described previously (9). Briefly, from May 2015 to August 2016, in Liaoning Medical University First affiliated Hospital, Jinzhou, China, a total of 71,020 inpatients were willing to pay fee for metabolomic profile measured. Of them, 1,032 consecutive patients with age over 18 years old and complete data in height, weight, and blood pressure were diagnosed as T2D. In the main analysis, we included 741 patients with complete information on fasting lipids including high-density lipoprotein cholesterol (HDL-C), low-density lipoprotein cholesterol (LDL-C), and triglyceride (TG). T2D was defined by the 1999 World Health Organization’s criteria (10) or as being treated with antidiabetic drugs. The Ethics Committee for Clinical Research of Liaoning Medical University First affiliated Hospital approved the ethics of the study, and informed consent was waivered due to the retrospective nature of the study, which is consistent with the Declaration of Helsinki.

### Data Collection

The retrieved data included the following information: demographic, anthropometric, current status of smoking and alcohol consumption, family history of CHD and stroke, duration of diabetes, clinical factors, drugs use and disease status. The clinical parameters included glycated hemoglobin (HbA1c), blood pressure (BP), HDL-C, LDL-C, and TG. The details regarding use of medications were documented, including use of oral antidiabetic drugs (acarbose, metformin, sulfonylureas, thiazolidinediones, and glinides), insulin, angiotensin-converting enzyme inhibitors (ACEIs) or angiotensin receptor blockers (ARBs), other antihypertensive drugs (calcium channel blocker, diuretics and beta-blockers), statins, other lipid lowering drugs, and aspirin.

### Clinical Definitions

CVD was defined as having history of CHD or stroke. CHD was defined as having a history of angina with abnormal electrocardiogram or on stress test, myocardial infarction, angina coronary artery bypass graft surgery or angioplasty; stroke was defined as nonfatal subarachnoid hemorrhage, intracerebral hemorrhage or other unspecified intracranial hemorrhage and ischemic stroke; Microvascular complication included diabetic retinopathy (DR) and diabetic nephropathy (DN). DR was defined as present if any of the following lesions was detected: microaneurysms, retinal hemorrhages, soft exudates, hard exudates, or vitreous hemorrhage. DN had following features: persistent albuminuria (or albuminuria excretion rate of >300 mg/d or 200 μg/min) recorded at least twice within a 3- to 6-month interval, progressive reduction in glomerular filtration rate (GFR) and hypertension. Body mass index (BMI) was calculated by weight in kilograms divided by squared height in meters. The World Health Organization criteria for Asians were used to classify BMI into four categories (11): underweight at <18 kg.m^−2^, normal weight at 18-24 kg.m^−2^, overweight at 24-28 kg.m^−2^, and obesity at >28kg.m^−2^. Hyperglycemia, high BP and abnormal lipids were defined as not reaching their treatment goals as recommended by American Diabetes Association, i.e., HbA1c ≥7% for hyperglycemia, BP ≥130/80 mmHg for high BP, TG ≥1.7 mmol/L, LDL-C ≥2.6 mmol/L or HDL-C ≥1 mmol/L in male and HDL-C ≥1.3 mmol/L in female for abnormal lipids (12).

### Laboratory Assays

Details of the metabolomics assessment method were published previously (13). Briefly, all blood sample was collected after 8-h of fasting and was stored as dried blood spot and used in the assay of metabolomics. A 3-mm (diameter) disc was punched from each dried blood spot paper. The discs were placed into the Millipore MultiScreen HV 96-well plate (Millipore, Billerica, MA, USA) for metabolite extraction. AB Sciex 4000 QTrap system (AB Sciex, Framingham, MA, USA) was used to conduct direct injection MS metabolomic analysis. An 80% acetonitrile aqueous solution was used as mobile phase. Analyst v1.6.0 software (AB Sciex) was used for system control and data collection. ChemoView 2.0.2 (AB Sciex) was used for data preprocessing. Isotope-labeled internal standard samples of 23 amino acids (NSK-A) were purchased from Cambridge Isotope Laboratories (Tewksbury, MA, USA), while standard samples of the amino acids were purchased from Chrom Systems (Grafelfing, Germany). Twenty-three amino acids were measured in our research, including alanine (Ala), asparagine (Asn), leucine (Leu), phenylalanine (Phe), tryptophan (Trp), tyrosine (Tyr), valine (Val), arginine (Arg), glycine (Gly), proline (Pro), threonine (Thr), citrulline (Cit), glutamine (Gln), histidine (His), lysine (Lys), methionine (Met), serine (Ser), ornithine (Orn), glutamate (Glu), aspartate (Asp), piperamide (Pip), cysteine (Cys), and homocysteine (Hcy).

### Statistical Analysis

Statistical analysis was conducted using SAS version 9.4 (SAS institute Inc., Cary, NC, USA) and R version 3.6.3. All P values were two tailed. P < 0.05 was considered to be statistically significant. Quantitative data with normal distribution were expressed as the mean ± standard deviation (SD) and data with non-normal distribution were presented as median with interquartile range (IQR). Normality was tested by checking Q-Q plot. Categorical data were presented as n(%). Student’s t-test if normal distribution was not rejected or Wilcoxon Two-Sample Test if normal distribution was rejected was used to compare the differences in continuous variables between CVD and non-CVD groups. The χ2 test was used to compare differences in categorical variables between the two groups.

Principal component analysis (PCA) was used to reduce the dimension of 23 AA. Orthogonal rotation (Varimax) was used to better interpret the results. Factors were retained according to eigenvalue, scree plot and variance (eigenvalue >1, gentle slope and adequate cumulative variance). Individual AA with absolute loadings >0.40 were considered to be the relevant components of the identified factors. Extracted factors were divided into tertiles when introduced in models.

A binary logistic regression was used to obtain odds ratio (OR) for CVD and 95% confidence intervals (CI) with a structured adjustment scheme. First, univariable analysis was used to obtain unadjusted OR, Then, stepwise analysis was performed to select factors independent of other extracted factors with the entry of factors which were statistically significant in univariate analysis (P < 0.05 for entry and exit). Third, multivariable logistic regression was conducted to evaluate OR of factors selected by stepwise regression with entry of traditional risk factors in step, i.e., age, sex, duration of diabetes, current smoking, current drinking, and BMI (≤18.5 kg/m^2^, 18.5–24 kg/m^2^, 24–28 kg/m^2^, ≥28 kg/m^2^) firstly; systemic blood pressure, HbA1c and HDL-C (<1mmol/L in male or <1.3mmol/L in female, ≥1mmol/L in male or ≥1.3 mmol/L in female), LDL-C (<2.6mmol/L, ≥2.6 mmol/L), and TG (<1.7mmol/L, ≥1.7mmol/L) secondly; antidiabetic drugs, lipid lowering drugs, and antihypertensive drugs lastly. Moreover, to compare effect on specific disease status, we further performed multivariable analysis for factors which were statistically significant in above-mentioned analysis to obtain OR for CHD or stroke alone. Associations between individual amino acids in significant factors and CVD (or its components) were also examined *via* same analysis procedure. Stratified analyses were also conducted to examine effects in different subgroups.

We compared the clinical characteristics by the factors tertiles. In this analysis, ANOVA (analysis of variance) or Kruskal-Wallis test where appropriate was used to compare the differences in continuous variables and the χ2 test was used to compare differences in categorical variables.

In the sensitivity analysis, we compared clinical characters of 1,032 subjects included and not included in our main analysis. PCA and logistic regression were repeated in these 1,032 T2D patients. Missing values of lipids were assigned as one category when introduced in multivariable model.

## Results

### Characteristics of the Study Patients

The 741 patients had a mean age of 57.9 (SD:14.1) years and median duration of T2D of 5 (IQR: 0–10) years. 52.8% Of them were male. The mean BMI of the cohort was 25.3 (SD: 3.8) kg/m^2^, with 43.3% of them being overweight and 20.8% being obese. Of the 741 patients, 282 (38.1%) had prior CVD (122 with CHD alone, 109 with stroke alone and 51 with both). 192 (25.9%) had microvascular disease. Compared with Non-CVD, patients with CVD were older and with longer duration of diabetes, SBP, lower LDL-C, lower HbA1c, lower insulin use, higher lipid lowering drugs use, higher antihypertensive drugs use, lower prevalence of DR. There were no difference in gender distribution, BMI, current smoking and drinking status, family history of CHD and stroke, DBP, HDL-C, TG, antidiabetic drugs use, and DN prevalence between two groups (**Table 1**). Arg, Asp, Cit, Cys, Glu, Hcy, His, Lys, Phe, and Pip were higher in patients with CVD. Other AA were similar in these two groups (**Table 2**).

**Table 1:** Clinical characteristics of patients with type 2 diabetes according to cardiovascular disease status.

Variables	Cross-sectional sample	CVD	Non-CVD	P-value*
	Mean±SD/N(%)	Mean±SD/N(%)	Mean±SD/N(%)	
N	741	282(X%)	459(X%)	
Age, years	57.9 ± 14.1	65.3 ± 11.1	53.3 ± 13.8	<.000
Duration of diabetes, years	5(0–10)	8(2–12)	4(0–10)	<.000
Male Gender	391(52.8%)	153(54.3%)	238(51.9%)	0.525
BMI, kg/m^2^	25.3 ± 3.8	25.1 ± 3.8	25.4 ± 3.7	0.423
BMI<18.5	17(2.3%)	9(3.2%)	8(1.7%)	0.283
BMI≥18.5 and <24	249(33.6%)	90(31.9%)	159(34.6%)	
BMI≥24 and <28	321(43.3%)	134(47.5%)	187(40.7%)	
BMI≥28	154(20.8%)	49(17.4%)	105(22.9%)	
Smoking		85(30.1%)	152(33.1%)	0.399
Drinking		69(24.5%)	136(29.6%)	0.127
Family history of CHD		9(3.2%)	14(3.1%)	0.620
Family history of stroke		10(3.6%)	8(1.7%)	0.122
SBP, mmHg		144.9 ± 24.6	137.1 ± 23.0	<.000
DBP, mmHg		83.1 ± 15.3	82.1 ± 12.5	0.344
hypertension		186(66.0%)	233(50.8%)	<.000
HDL-C, mmol/L		1.08 ± 0.35	1.09 ± 0.35	0.772
<1 in male or <1.3 in female		97(34.4%)	150(32.7%)	0.630
≥1 in male or ≥1.3 in female		185(65.6%)	309(67.3%)	
LDL-C, mmol/L		2.75 ± 0.90	2.98 ± 1.07	0.002
<2.6		132(46.8%)	175(38.1%)	0.020
≥2.6		150(53.2%)	284(61.9%)	
Triglyceride, mmol/L		1.62(1.09–2.22)	1.69(1.12–2.60)	0.069
<1.7		152(53.9%)	230(50.1%)	0.316
≥1.7		130(46.1%)	229(49.9%)	
HbA1c, %		8.93 ± 2.31	9.87 ± 2.32	<.000
<7		32(11.4%)	34(7.4%)	<.000
7-8		46(16.3%)	50(10.9%)	
≥8		112(39.7%)	267(58.2%)	
Lack		92(32.6%)	108(23.5%)	
Antidiabetic drugs		234(83.0%)	403(87.8%)	0.067
OAD		147(52.1%)	281(61.2%)	0.015
Insulin		189(67.0%)	376(81.9%)	<.000
Lipid lowering drugs		162(57.5%)	157(34.2%)	<.000
Statins		160(56.7%)	146(31.8%)	<.000
OLLD		3(1.1%)	15(3.3%)	0.059
Antihypertensive drugs		178(63.1%)	158(34.4%)	<.000
Angiotensin drugs		107(37.9%)	103(22.4%)	<.000
OAHD		150(53.2%)	102(22.2%)	<.000
Diabetic nephropathy	145(19.6%)	50(17.7%)	95(20.7%)	0.323
Diabetic retinopathy	94(12.7%)	24(8.5%)	70(15.3%)	0.007
CHD alone	122			
Stroke alone	109			
Both CHD and stroke	51			

*P-value was acquired by comparing CVD and Non-CVD.

SD, standard deviation; IQR, interquartile range; N, number, CVD, cardiovascular disease; Non-CVD, without cardiovascular disease; SD, standard deviation; IQR, interquartile range; BMI, body mass index; SBP, systolic blood pressure; DBP, diastolic blood pressure; HDL-C, high-density lipoprotein cholesterol; LDL-C, low-density lipoprotein cholesterol; HbA1c, glycated hemoglobin; OAD, oral antidiabetic drugs; OLLD, other lipid lowering drugs; OAHD, other antihypertensive drugs; CHD, coronary heart disease.

Data are mean (SD), median (IQR), or n (%).

P values were derived from Chi-square test or Student t test.

**Table 2:** Amino acid profile and identified factors by CVD status.

Variables	CVD	Non-CVD	P-value
	Mean ± SD/N(%)	Mean ± SD/N(%)	
Ala, μmol/L	131.7 ± 41.6	129.5 ± 46.4	0.514
Arg, μmol/L	13.8 ± 9.4	11.6 ± 9.2	0.002
Asn, μmol/L	76.2 ± 22.1	79.3 ± 24.1	0.085
Asp, μmol/L	32.0 ± 13.4	29.7 ± 12.1	0.017
Cit, μmol/L	23.1 ± 12.3	20.8 ± 8.7	0.006
Cys, μmol/L	1.4(1.0–1.8)	1.2(1.2–1.7)	0.014
Gln, μmol/L	7.2(5.0–9.7)	6.7(5.1–9.1)	0.292
Glu, μmol/L	109.7 ± 36.4	103.7 ± 36.8	0.031
Gly, μmol/L	218.3 ± 90.7	214.2 ± 88.4	0.542
Hcy, μmol/L	7.7 ± 1.0	7.6 ± 1.1	0.043
His, μmol/L	57.9(35.9–87.6)	49.0(34.2–76.9)	0.022
Leu, μmol/L	130.9 ± 42.4	136.8 ± 47.5	0.078
Lys, μmol/L	150.9 ± 96.7	135.8 ± 72.2	0.024
Met, μmol/L	18.1 ± 7.0	18.8 ± 6.6	0.180
Orn, μmol/L	17.4(13.4–23.7)	17.8(13.0–23.6)	0.925
Phe, μmol/L	49.9 ± 17.0	47.4 ± 15.6	0.048
Pip, μmol/L	135. 6(102.0–186.0)	121.4(90.8–169.4)	0.003
Pro, μmol/L	473.2 ± 202.2	481.9 ± 206.4	0.571
Ser, μmol/L	56.4 ± 22.9	56.7 ± 19.9	0.875
Thr, μmol/L	26.6 ± 10.5	26.1 ± 9.3	0.512
Trp, μmol/L	49.2 ± 13.5	48.4 ± 14.7	0.453
Tyr, μmol/L	47.6 ± 16.4	49.4 ± 17.5	0.169
Val, μmol/L	140.5 ± 39.3	142.2 ± 41.5	0.565
Factor 1			0.078
<−0.48	106(37.6%)	141(30.7%)	
−0.48–0.25	94(33.3 %)	152(33.1 %)	
>0.25	82(29.1%)	166(36.2%)	
Factor 2			0.2342
<−0.38	89(31.6%)	158(34.4%)	
−0.38–0.11	88(31.2%)	158(34.4%)	
>0.11	105(37.2%)	143(31.2%)	
Factor 3			0.0208
<−0.47	81(28.7%)	165(36.0%)	
−0.47–0.14	90(31.9%)	157(34.2%)	
>0.14	111(39.4%)	137(29.9%)	
Factor 4			0.0155
<−0.55	77(27.3%)	170(37.0%)	
−0.55–0.27	97(34.4%)	149(32.5%)	
>0.27	108(38.3%)	140(30.5%)	
Factor 5			0.1791
<−0.47	105(37.2%)	141(30.7%)	
−0.47–0.13	90(31.9%)	157(34.2%)	
>0.13	87(30.9%)	161(35.1%)	

CVD, cardiovascular disease; Non-CVD, without cardiovascular disease; SD, standard deviation; IQR, interquartile range; N, number; Ala, Alanine; Asn, Asparagine; Leu, Leucine; Phe, Phenylalanine; Trp, Tryptophan; Tyr, Tyrosine; Val, Valine; Arg, Arginine; Gly, Glycine; Pro, Proline; Thr, Threonine; Cit, Citrulline; Gln, Glutamine; His, Histidine; Lys, Lysine; Met, Methionine; Ser, Serine; Orn, Ornithine; Glu, Glutamate; Asp, aspartate; Pip, Piperamide, Cys, Cysteine; Hcy, Homocysteine.

Factor 1 included Asn, Leu, Phe, Tyr, and Val.

Factor 2 included Gln, His, and Lys.

Factor 3 included Glu and Trp.

Factor 4 included Arg.

Factor 5 included Ser.

### Extracted Factors of Amino Acids

Five factors were extracted, explaining 65% of the total variance. Factor 1 mainly included branched chain and aromatic amino acids, i.e., Asn, Leu, Phe, Tyr, and Val; Factor 2 included Gln, His, and Lys; Factor 3 included Glu and Trp; Factor 4 included Arg; Factor 5 included Ser. Loadings were bold when they were larger then 0.40; Asn, Leu and Val in factor 1, Gln and Lys in factor 2, Glu in factor 3, Arg in factor 4 and Ser in factor 5 had loadings over 0.80 (**Table 3**). Higher tertiles of factor3 and factor 4 (factor 3 > 0.14, factor 4 ≥ −0.55) were more frequent in CVD than non-CVD (**Table 2**).

**Table 3:** The factors extracted from the 23 amino acids and their loadings.

	Factor 1	Factor 2	Factor 3	Factor 4	Factor 5
Ala	0.32	0.13	0.18	0.10	0.09
Arg	0.17	0.06	0.06	**0.93**	0.05
Asn	**0.92**	0.05	0.09	0.07	0.09
Asp	0.15	0.06	0.22	0.06	0.17
Cit	0.09	0.12	0.13	0.15	0.12
Cys	0.04	0.07	−0.00	−0.01	0.02
Gln	0.05	**0.90**	0.10	−0.04	0.00
Glu	0.17	0.14	**0.86**	0.04	0.15
Gly	0.11	0.03	0.18	0.21	0.22
Hcy	−0.05	0.00	−0.01	0.01	0.03
His	0.10	**0.46**	0.08	0.13	0.12
Leu	**0.89**	0.03	0.11	0.08	0.12
Lys	0.04	**0.92**	0.06	0.10	0.01
Met	0.39	0.39	0.07	0.08	0.09
Orn	0.00	0.06	0.15	0.01	0.07
Phe	**0.44**	0.03	0.15	0.12	0.11
Pip	0.05	0.11	0.08	0.02	0.02
Pro	0.26	0.16	0.05	0.13	0.04
Ser	0.21	0.02	0.17	0.06	**0.87**
Thr	0.31	0.12	0.18	0.21	0.24
Trp	0.37	0.14	**0.53**	0.20	0.20
Tyr	**0.52**	0.05	0.15	0.12	0.21
Val	**0.90**	0.04	0.05	0.09	0.03
eigenvalue	8.20	2.28	1.91	1.33	1.14
CV	0.36	0.46	0.54	0.60	0.65

Displayed are the five metabolomic factors identified by principal component analysis and corresponding eigenvalue and cumulative variance. Number of identified factors was decided synthetically according to eigenvalue > 1, scree plot, and cumulative variance. Individual amino acid with absolute loading >0.40 was considered to be the relevant component of the identified factors.

Ala, Alanine; Asn, Asparagine; Leu, Leucine; Phe, Phenylalanine; Trp, Tryptophan; Tyr, Tyrosine; Val, Valine; Arg, Arginine; Gly, Glycine; Pro, Proline; Thr, Threonine; Cit, Citrulline; Gln, Glutamine; His, Histidine; Lys, Lysine; Met, Methionine; Ser, Serine; Orn, Ornithine; Glu, Glutamate; Asp, aspartate; Pip, Piperamide, Cys, Cysteine; Hcy, Homocysteine.

### Association Between Extracted Factors and CVD Risk in T2D

**Figure 1:**
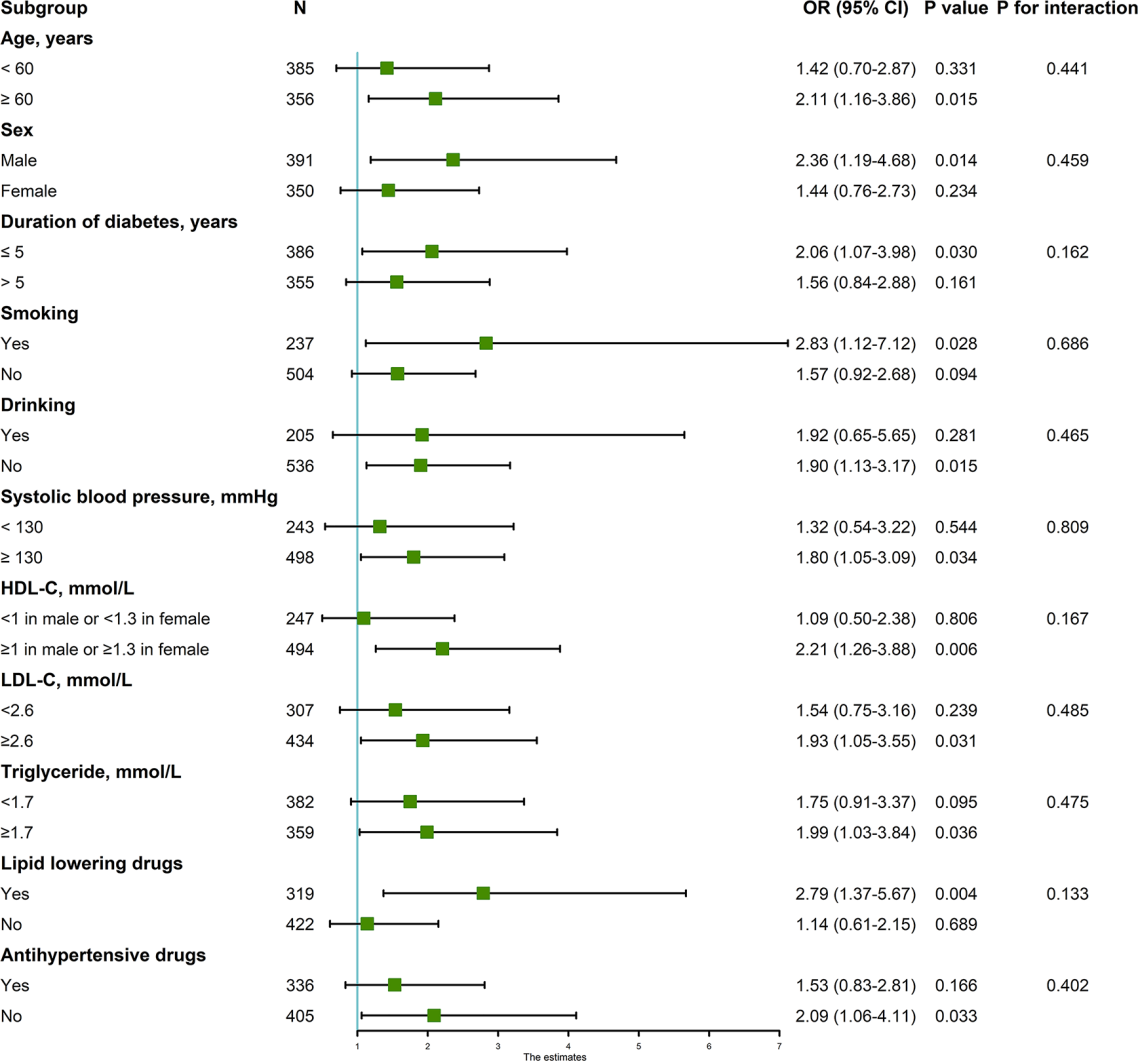
Associations between factor3 (top tertile vs. bottom tertile) and CVD in subgroups. Adjusted for age, sex, duration of diabetes, current smoking, current drinking, body mass index (≤18.5 kg/m^2^, 18.5–24 kg/m^2^, 24–28 kg/m^2^, ≥28 kg/m^2^), systolic blood pressure, high-density lipoprotein cholesterol (<1 mmol/L in male or <1.3 mmol/L in female, ≥1 mmol/L in male or ≥1.3 mmol/L in female), low-density lipoprotein cholesterol (<2.6 mmol/L, ≥2.6 mmol/L), glycated hemoglobin (<7%, 7%~8%, ≥8%, lack), triglyceride (<1.7 mmol/L, ≥1.7 mmol/L), antidiabetic drugs, lipid lowering drugs, and antihypertensive drugs. CVD, cardiovascular diseases; OR, odds ratio; CI, confidence interval; BMI, body mass index; SBP, systolic blood pressure; DBP, diastolic blood pressure; HDL-C, high-density lipoprotein cholesterol; LDL-C, low-density lipoprotein cholesterol; HbA1c, glycated hemoglobin.

In univariable analysis, factor 1, factor 3, and factor 4 were associated with CVD risk but only factor 3 (OR, 95%CI: top tertile vs. bottom tertile 1.67 (1.15–2.41), P for trend = 0.021) and factor 4 (OR, 95%CI: top tertile vs. bottom tertile 1.70 (1.18–2.46), P for trend = 0.016) were retained after stepwise regression (**Table 4**); After adjusted for traditional risk factors, only factor 3 was still predictive of CVD with OR slightly changing (OR, 95%CI: top tertile vs. bottom tertile 1.77 (1.13–2.76), P for trend = 0.012) (**Table 5**); besides, the direction of this association were the same in all subgroups, although not all effects were statistically significant. There were no interaction between each pair subgroups (**Figure 1**); when defining CHD alone or stroke alone as endpoint, factor 3 was only steadily associated with stroke but not CHD. The OR for stroke was increased comparing with OR for CVD (adjusted OR, 95%CI: top tertile vs. bottom tertile 2.19 (1.17–4.07), P for trend = 0.014) (**Table 6**).

**Table 4:** Odds ratio of extracted factors for CVD risk in T2D.

	OR(95%CI)	P-value	P for trend
**Univariate analysis**			
Factor 1			0.024
<−0.48	1.00		
−0.48–0.25	0.82(0.57–1.18)	0.288	
≥0.25	0.66(0.46–0.95)	0.024	
Factor 2			0.149
<−0.38	1.00		
−0.38–0.11	0.99(0.68–1.43)	0.952	
≥0.11	1.30(0.91–1.88)	0.151	
Factor 3			0.007
<−0.47	1.00		
−0.47–0.14	1.17 (0.81–1.69)	0.413	
≥0.14	1.65 (1.15–2.38)	0.007	
Factor 4			0.005
<−0.55	1.00		
−0.55–0.27	1.44(0.99–2.08)	0.056	
≥0.27	1.70(1.18–2.46)	0.005	
Factor 5			0.083
<−0.47	1.00		
−0.47–0.13	0.77(0.54–1.11)	0.157	
≥0.13	0.73(0.51–1.04)	0.083	
**Stepwise analysis**			
Factor 3			0.021
<−0.47	1.00		
−0.47–0.14	1.16(0.80–1.68)	0.443	
≥0.14	1.67(1.15–2.41)	0.007	
Factor 4			0.016
<−0.55	1.00		
−0.55–0.27	1.50(1.03–2.18)	0.034	
≥0.27	1.70(1.18–2.46)	0.005	

CVD, cardiovascular disease; OR, odds ratio; CI, confidence interval.

Stepwise analysis was performed to select factors with the entry of factors which were statistically significant in univariate analysis (P < 0.05 for entry and exit).

**Table 5:** Adjusted odds ratio of factors for CVD risk in T2D.

	OR(95%CI)	P-value	P for trend
**Model 1**			
Factor 3			0.013
<−0.47	1.00		
−0.47–0.14	1.04(0.69–1.59)	0.845	
≥0.14	1.68(1.11–2.55)	0.014	
Factor 4			0.111
<−0.55	1.00		
−0.55–0.27	1.38(0.91–2.09)	0.133	
≥0.27	1.41(0.93–2.13)	0.109	
**Model 2**			
Factor 3			0.010
<−0.47	1.00		
−0.47–0.14	1.06(0.69–1.63)	0.786	
≥0.14	1.73(1.13–2.65)	0.011	
Factor 4			0.207
<−0.55	1.00		
−0.55–0.27	1.40(0.92–2.14)	0.117	
≥0.27	1.32(0.86–2.01)	0.204	
**Model 3**			
Factor 3			0.012
<−0.47	1.00		
−0.47–0.14	1.16(0.74–1.81)	0.512	
≥0.14	1.77(1.13–2.76)	0.012	
Factor 4			0.453
<−0.55	1.00		
−0.55–0.27	1.42(0.91–2.21)	0.119	
≥0.27	1.18(0.76–1.84)	0.456	

CVD, cardiovascular disease; OR, odds ratio; CI, confidence interval.

Model 1, adjusted for age, sex, duration of diabetes, current smoking, current drinking, body mass index (≤18.5 kg/m^2^, 18.5–24 kg/m^2^, 24–28 kg/m^2^, ≥28 kg/m^2^).

Model 2, adjusted for variables in model 1 plus systolic blood pressure, high-density lipoprotein cholesterol (<1 mmol/L in male or <1.3 mmol/L in female, ≥1 mmol/L in male or ≥1.3 mmol/L in female), low-density lipoprotein cholesterol (<2.6 mmol/L, ≥2.6 mmol/L), glycated hemoglobin (<7%, 7%~8%, ≥8%, lack), triglyceride (<1.7 mmol/L, ≥1.7 mmol/L).

Model 3, adjusted for variables in model 2 plus antidiabetic drugs, lipid lowering drugs, and antihypertensive drugs.

**Table 6:** Odds ratio of factor 3 and individual amino acids for CHD or stroke risk in T2D.

	OR(95%CI)	P-value	P for trend
**OR of CHD risk in T2D(122/581)**			
Factor 3			0.178
<−0.47	1.00		
−0.47–0.14	1.39(0.76–2.52)	0.283	
≥0.14	1.51(0.83–2.73)	0.177	
Glu, μmol/L			0.230
<89.3	1.00		
89.3–111.2	1.10(0.61–1.98)	0.751	
≥111.2	1.44(0.80–2.61)	0.230	
Trp, μmol/L			
<41.7	1.00		0.835
41.7–53.5	1.14(0.63–2.07)	0.670	
≥53.5	1.07(0.58–1.97)	0.827	
**OR of stroke risk in T2D(109/568)**			
Factor 3			0.014
<−0.47	1.00		
−0.47–0.14	1.62(0.86–3.03)	0.134	
≥0.14	2.19(1.17–4.07)	0.014	
Glu, μmol/L			0.007
< 89.3	1.00		
89.3-111.2	1.29(0.64–2.60)	0.554	
≥111.2	2.62(1.18–5.84)	0.007	
Trp, μmol/L			0.197
<41.7	1.00		
41.7–53.5	1.27(0.68–2.35)	0.452	
≥53.5	1.50(0.81–2.75)	0.196	

CHD, coronary heart disease; OR, odds ratio; CI, confidence interval; Glu, glutamate; Trp, tryptophan.

122/581, 122 patients with CHD in 581 T2D patients without stroke. 109/568, 109 patients with stroke in 568 T2D patients without CHD.

Adjusted for age, sex, duration of diabetes, current smoking, current drinking, body mass index (≤18.5 kg/m^2^, 18.5–24 kg/m^2^, 24–28 kg/m^2^, ≥28 kg/m^2^), systolic blood pressure, high-density lipoprotein cholesterol (<1 mmol/L in male or <1.3 mmol/L in female, ≥1 mmol/L in male or ≥1.3 mmol/L in female), low-density lipoprotein cholesterol (<2.6 mmol/L, ≥2.6 mmol/L), glycated hemoglobin (<7%, 7%~8%, ≥8%, lack), triglyceride (<1.7 mmol/L, ≥1.7 mmol/L), antidiabetic drugs, lipid lowering drugs, and antihypertensive drugs.

### Association Between Individual Amino Acids in Factor 3 and CVD Risk in T2D

Glu was positively associated with stroke (OR, 95%CI: top tertile vs. bottom tertile 2.62 (1.18–5.84), P for trend = 0.007) but not CHD (OR, 95%CI: top tertile vs. bottom tertile: 1.44 (0.80–2.61), P for trend = 0.230); Trp was not significantly associated with CHD or stroke (**Table 6**).

### General Characteristics Across Tertiles of Factor 3

Higher level of factor 3 were more frequent in obesity. Other characteristics were similar among categories of factor 3 (**Table 7**).

**Table 7:** Clinical characters according to factor 3 classification.

Variables	<−0.47	−0.47–0.14	>0.14	P-value^#^	
	Mean ± SD/N(%)	Mean ± SD/N(%)	Mean ± SD/N(%)	
Age	56.4 ± 14.7	58.8 ± 13.3	58.4 ± 14.0	0.107	
SBP, mmHg	138.5 ± 24.4	140.2 ± 22.1	141.5 ± 25.0	0.387	
DBP, mmHg	82.9 ± 14.2	82.2 ± 13.0	82.3 ± 13.8	0.840	
Duration of diabetes	5(0-10)	5(0-11)	6(1-10)	0.460	
Sex(male)	138(56.1%)	125(50.6%)	128(51.6%)	0.430	
HbA1c, %	9.61 ± 2.45	9.34 ± 2.19	9.67 ± 2.42	0.352	
<7	24(9.8%)	22(8.9%)	20(8.1%)	0.789	
7-8	31(12.6%)	36(14.6%)	29(11.7%)		
≥8	132(53.7%)	121(49.0%)	126(50.8%)		
Lack	59(24.0%)	68(27.5%)	73(29.4%)		
HDL-C, mmol/L	1.09 ± 0.36	1.07 ± 0.32	1.08 ± 0.36	0.779	
<1 in male or <1.3 in female	85(34.6%)	74(30.0%)	88(35.5%)	0.378	
≥1 in male or ≥1.3 in female	161(65.5%)	173(70.0%)	160(64.5%)		
LDL-C, mmol/L	2.83 ± 1.02	2.92 ± 1.01	2.92 ± 1.00	0.486	
<2.6	111(45.1%)	98(39.7%)	98(39.5%)	0.355	
≥2.6	135(54.9%)	149(60.3%)	150(60.5%)		
Triglyceride, mmol/L	1.58(1.02–2.29)	1.63(1.14–2.38)	1.78(1.13–2.50)	0.213	
<1.7	135(54.9%)	132(53.4%)	115(46.4%)	0.128	
≥1.7	111(45.1%)	115(46.6%)	133(53.6%)		
BMI, kg/m^2^	24.9 ± 3.6	25.3 ± 3.7	25.6 ± 4.00	0.138	
BMI < 18.5	6(2.4%)	5(2.0%)	6(2.4%)	0.003	
BMI ≥ 18.5 and <24	88(35.8%)	81(32.8%)	80(32.3%)		
BMI ≥ 24 and <28	111(45.1%)	108(43.7%)	102(41.1%)		
BMI ≥ 28	41(16.7%)	53(21.5%)	60(24.2%)		

SD, standard deviation; IQR, interquartile range; N, number; BMI, body mass index; SBP, systolic blood pressure; DBP, diastolic blood pressure; HbA1c, glycated hemoglobin; HDL-C, high-density lipoprotein cholesterol; LDL-C, low-density lipoprotein cholesterol.

Data are mean (SD), median (IQR), or n (%).

^#^P values were derived from ANOVA (analysis of variance) or Kruskal-Wallis test or Chi-Square test.

### Validation

When compared patients included and not included in our main analysis, patients in the second data set were younger and with lower rate of CVD, DN, DR and use of antidiabetic drugs, lipid lowering drugs, and antihypertensive drugs. Patients in the second data set were with healthier status (**Table S1**). The results of PCA in total 1,032 patients were the same with results in main analysis (**Table S2**). The associations between factor 3 and individual amino acids with CVD risk were consistence with associations in main analysis (**Table S3**).

## Discussion

Metabolomics is emerging as a useful tool for identifying novel biomarkers of diseases (14). In current study, we explored global patterns of diverse PFAA and their associations with CVD risk in T2D cohort in China so as to elucidate potential mechanisms involved in CVD progression among Chinese diabetic patients. The key finding of our study was that factor 3 mostly composed of Glu and Trp was positively associated with CVD. Furthermore, the association was more pronounced for stroke and the risk association was decreased to non-significance for CHD. When it comes to individual amino acid, the relationships between Glu and CVD (or its components) were the same with the relationships between factor 3 and CVD (or its components). However, Trp was not associated with either CHD or stroke significantly.

Glu is one of the most common non-essential amino acids. Homeostasis of Gln and Glu was essential for various functions such as insulin section, gluconeogenesis, and glutathione synthesis (15). Role of Glu in metabolic disturbance and diseases has been investigated in several previous epidemiological research. The early Framingham Heart Study had revealed a positive correlation between Glu levels and insulin resistance traits in general population (16). Consistently, several prospective studies found that Glu was positively associated with CVD (17, 18), and especially stroke (17). However, all these research were conducted in general population rather than diabetic group, who are more likely to accompany with and die from advanced CVDs. Besides, none of them were in Chinese population. In the current Chinese diabetic group, we found that Glu was positively associated with CVD, especially stroke, but not CHD. The risk association between Glu and stroke may be due to its role in thrombogenesis because numbers of studies found that Glu is involved in platelet activation and thrombogenesis (19). Discrepant finding regarding to CHD between our and previous study (18) may derive from two possible reasons: 1) linkages between Glu and CHD are different in general and diabetic population; 2) heterogeneity of CHD subtypes, i.e., proportion of ischemic myocardial infarction, may contribute to the discrepancy. According to our hypothesis, Glu may relate to CHD subtypes with thrombogenesis. Future investigations were needed to understand role of Glu in thrombogenesis in T2D.

Trp is an essential amino acid and can be metabolized to downstream catabolites such as kynurenine, kynurenic acid, 3-hydroxyanthranilic acid and so on (20). Recently Trp, especially its catabolites, were found to be related to CVD and its risk factors (20–22). A prospective study in general population revealed that increased levels of baseline plasma Trp were significantly associated with decreased risk of myocardial infarction and coronary artery disease death (23). Inconsistently, in the present study, we did not found a significant relationship between plasma Trp and CVD or its components in this diabetic group, although the direction was positive. The discrepancy may derive from heterogeneity of general and diabetic population. In general population, accelerated Trp degradation led to lower Trp and higher catabolites concentration, which resulted in elevated CVD risk; in patients with T2D, both Trp and its catabolized products were increased than healthy subjects (24, 25), suggesting more substrate and metabolites harmful to cardiovascular metabolism in individual with T2D (26). Investigations involving Trp metabolites are warranted.

Given to the closer relationship of factor 3 and thrombosis-related diseases in T2D, we speculated that crosstalk between Glu and Trp was essential for thrombus formation at advanced atherosclerosis. Experiment on mice identified Glu as regulator of platelet activation *via* binding to the adenosine monophosphate-activated protein kinase receptor to increase intracellular sodium concentration and depolarize platelets (19). Trp catabolites were more frequently seen in unstable fibrous plaques rather than stable plaques (27) and can mediate platelet adhesion in vessel injury (28–30). Therefore, elevated plasma Glu and Trp might promote platelet activation and adhesion, respectively, contributing to final thrombogenesis in T2D.

Our findings had important potential public health and clinical implications. T2D is one of the most common public and clinical health issue. Thrombosis is one of the severe complications in T2D patients. Our findings provided a new target of myocardial infarction and ischemic stroke prevention in T2D patients for future research on their possible role in CVD in T2D. Our study had several limitations. First, our study was a cross-sectional survey and we did not have information on duration of CVD, so the causal relationships could not be established; second, we did not collect information on lifestyle factors like diet and physical activity. BMI may account for partial effects of lifestyle factors on diseases and adjustment for BMI may remove partial confounding effect of lifestyle factors; third, we did not collect information on socioeconomic variables (education and income). Patients who were willing to pay for metabolic profile measuring in our study may have better education and income. Future investigations should include subjects with diverse characters; fourth, 291 diabetic patients without data on lipids had healthier body status. When include them in analysis, the associations between metabolites and CVD were only slightly attenuated, suggesting robust findings in our study; fifth, we did not document the subtypes of stroke and CHD and their associations with individual components of CHD and stroke were unknown. Finally, we did not measure Trp catabolites and other molecules involved in thrombogenesis, future research should include these molecules to better interpret pathogenesis of CVD in T2D.

In conclusion, we found that factor 3 composed of Glu and Trp was associated with increased risk of CVD especially stroke in hospital-based Chinese T2D patients. More high quality epidemiological and experimental studies are warranted to confirm and explain our findings.

## Data Availability Statement

The datasets generated for this study can be found in Metabolights, https://www.ebi.ac.uk/metabolights/search, with the accession number MTBLS1427.

## Ethics Statement

The studies involving human participants were reviewed and approved by The Ethics Committee for Clinical Research of Liaoning Medical University First affiliated Hospital. The ethics committee waived the requirement of written informed consent for participation.

## Author Contributions

J-LW and G-GW designed the study. TL and H-HL analyzed the data and wrote the draft. X-FF and Z-ZF gave critical comments on the writing. YB gave critical comments on metabolomics. All authors contributed to the article and approved the submitted version.

## Funding

TThis study was supported by the project for the National Key Research and Development Program of China (2019YFA0802300), The 13th five year plan and TMU talent project (11601501/2016KJ0313), National Natural Science Foundation of China (No. 81602826, 81672961), the China Postdoctoral Science Foundation (2016M590210, 2017T100164), Tianjin Health Bureau Science Foundation Key Project (16KG154), Tianjin Project of Thousand Youth Talents, Key Laboratory Open Project Fund from State Key Laboratory of Environmental Chemistry and Ecotoxicology, Research Center for Eco-Environmental Sciences, Chinese Academy of Sciences (KF2017), Postgraduate Innovation Fund of “13th Five-Year comprehensive investment”, Tianjin Medical University (YJSCX201816), and The Open Project of the Key Laboratory of Modern Toxicology of Ministry of Education, Nanjing Medical University (NMUMT201809).

## Conflict of Interest

The authors declare that the research was conducted in the absence of any commercial or financial relationships that could be construed as a potential conflict of interest.
