# Supplementary material for: Plasma Free Amino Acids and Risk of Cardiovascular Disease in Chinese Patients With Type 2 Diabetes
Source: Front Endocrinol (Lausanne). 2021 Apr 14;11:519923. doi: 10.3389/fendo.2020.519923 (PMC8081348; doi:10.3389/fendo.2020.519923)
Supplement: Supplementary file 1 [file Table_1.doc]

Table S1. Comparison of additional data set of the T2D patients not included in the main analysis

| Variable | Included | Not included | P-value |
| --- | --- | --- | --- |
| N | 741 | 291 |  |
| CVD | 282 (38.1%) | 68 (23.4%) | <.001 |
| Age, years | 57.9 ± 14.1 | 55.6 ± 13.0 | 0.015 |
| Duration of diabetes, years | 5(0-10) | 4 (0-10) | 0.0942 |
| Male Gender | 391(52.8%) | 158 (54.3%) | 0.658 |
| BMI, kg/m2 | 25.3±3.8 | 25.3 ± 4.1 | 0.874 |
| BMI<18.5 | 17(2.3%) | 10 (3.4%) | 0.304 |
| BMI≥18.5 and <24 | 249(33.6%) | 105 (36.1%) |  |
| BMI≥24 and <28 | 321(43.3%) | 109 (37.5%) |  |
| BMI≥28 | 154(20.8%) | 67 (23.0%) |  |
| Smoking | 237 (32.0%) | 94 (32.3%) | 0.921 |
| Drinking | 205 (27.7%) | 85 (29.2%) | 0.620 |
| Family history of CHD | 23 (3.1%) | 3 (1.0%) | 0.075 |
| Family history of stroke | 18 (2.4%) | 6 (2.1%) | 0.822 |
| SBP, mmHg | 140.1 ± 23.9 | 141.3 ± 24.3 | 0.467 |
| DBP, mmHg | 82.5 ± 13.6 | 82.5 ± 13.2 | 0.994 |
| HDL-C, mmol/L | 1.08 ± 0.35 |  |  |
| LDL-C, mmol/L | 2.89 ± 1.01 |  |  |
| Triglyceride, mmol/L | 1.67 (1.11-2.38) |  |  |
| HbA1c, % | 9.54 ± 2.36 |  |  |
| Antidiabetic drugs | 637 (86.0%) | 230 (79.0%) | 0.006 |
| Lipid lowering drugs | 319 (43.1%) | 69 (23.7%) | <.001 |
| Antihypertensive drugs | 336 (45.3%) | 77 (26.5%) | <.001 |
| Diabetic nephropathy | 145 (19.6%) | 43 (14.8%) | 0.073 |
| Diabetic retinopathy | 94 (12.7%) | 68 (23.4%) | <.001 |

Abbreviations: N, number, CVD, cardiovascular disease; SD, standard deviation; IQR, interquartile range; BMI, body mass index; SBP, systolic blood pressure; DBP, diastolic blood pressure; HDL-C, high-density lipoprotein cholesterol; LDL-C, low-density lipoprotein cholesterol; HbA1c, glycated hemoglobin;

Data are mean (standard deviation), median (interquartile range), or n (%);

P values were derived from Chi-square test (or fisher test) or Student t test (or Wilcoxon Two-Sample Test).

Table S2. The factors extracted from the 23 amino acids and their loadings in 1032 patients

|  | Factor 1 | Factor 2 | Factor 3 | Factor 4 | Factor 5 |
| --- | --- | --- | --- | --- | --- |
| Ala | 0.32 | 0.12 | 0.17 | 0.11 | 0.08 |
| Arg | 0.18 | 0.08 | 0.06 | **0.92** | 0.06 |
| Asn | **0.93** | 0.03 | 0.09 | 0.07 | 0.09 |
| Asp | 0.16 | 0.06 | 0.21 | 0.06 | 0.19 |
| Cit | 0.07 | 0.12 | 0.14 | 0.16 | 0.11 |
| Cys | 0.06 | 0.06 | 0.00 | -0.01 | 0.01 |
| Gln | 0.04 | **0.89** | 0.10 | -0.03 | -0.01 |
| Glu | 0.15 | 0.12 | **0.88** | 0.03 | 0.12 |
| Gly | 0.13 | 0.02 | 0.21 | 0.20 | 0.21 |
| Hcy | -0.05 | 0.03 | -0.01 | 0.01 | 0.02 |
| His | 0.09 | **0.43** | 0.10 | 0.13 | 0.13 |
| Leu | **0.89** | 0.03 | 0.12 | 0.09 | 0.11 |
| Lys | 0.03 | **0.91** | 0.05 | 0.12 | 0.00 |
| Met | 0.40 | 0.32 | 0.08 | 0.08 | 0.11 |
| Orn | 0.00 | 0.06 | 0.16 | 0.01 | 0.06 |
| Phe | **0.39** | 0.01 | 0.16 | 0.10 | 0.14 |
| Pip | 0.06 | 0.06 | 0.07 | 0.03 | 0.03 |
| Pro | 0.26 | 0.16 | 0.05 | 0.13 | 0.03 |
| Ser | 0.23 | -0.00 | 0.17 | 0.06 | **0.87** |
| Thr | 0.33 | 0.11 | 0.16 | 0.21 | 0.24 |
| Trp | 0.35 | 0.13 | **0.58** | 0.18 | 0.18 |
| Tyr | **0.49** | 0.04 | 0.16 | 0.11 | 0.20 |
| Val | **0.89** | 0.04 | 0.05 | 0.10 | 0.06 |
| eigenvalue | 8.24 | 2.28 | 1.85 | 1.25 | 1.14 |
| CV | 0.36 | 0.46 | 0.54 | 0.59 | 0.64 |

Displayed are the 5 metabolomic factors identified by principal component analysis and corresponding eigenvalue and cumulative variance. Number of identified factors was decided synthetically according to eigenvalue>1,scree plot and cumulative variance. Individual amino acid with absolute loading >0.40 was considered to be the relevant component of the identified factors.

Abbreviations: Ala, Alanine; Asn, Asparagine; Leu, Leucine; Phe, Phenylalanine; Trp, Tryptophan; Tyr, Tyrosine; Val, Valine; Arg, Arginine; Gly, Glycine; Pro, Proline; Thr, Threonine ; Cit , Citrulline; Gln, Glutamine; His, Histidine; Lys, Lysine; Met, Methionine; Ser, Serine; Orn, Ornithine; Glu, Glutamate; Asp, aspartate; Pip，Piperamide, Cys, Cysteine; Hcy, Homocysteine.

Table S3. Odds ratio of factor 3 and individual amino acids for CVD risk in 1032 patients with T2D

|  | OR(95%CI) | P-value | P for trend |
| --- | --- | --- | --- |
| **Univariable model** |  |  |  |
| Factor 3 |  |  | 0.005 |
| <-0.48 | 1.00 |  |  |
| -0.48-0.16 | 1.05 (0.76-1.45) | 0.785 |  |
| ≥0.16 | 1.56 (1.14-2.13) | 0.006 |  |
| Glu, μmol/L |  |  | 0.002 |
| < 87.5 | 1.00 |  |  |
| 87.5-111.1 | 1.34 (0.97-1.86) | 0.074 |  |
| ≥ 111.1 | 1.67 (1.22-2.31) | 0.002 |  |
| Trp, μmol/L |  |  |  |
| < 41.6 | 1.00 |  | 0.034 |
| 41.6-53.4 | 1.25 (0.91-1.72) | 0.174 |  |
| ≥ 53.4 | 1.41 (1.03-1.94) | 0.033 |  |
| **Multivariable model** |  |  |  |
| Factor 3 |  |  | 0.048 |
| <-0.48 | 1.00 |  |  |
| -0.48-0.16 | 1.10 (0.75-1.60) | 0.638 |  |
| ≥0.16 | 1.46 (1.00-2.12) | 0.049 |  |
| Glu, μmol/L |  |  | 0.040 |
| < 87.5 | 1.00 |  |  |
| 87.5-111.1 | 1.19 (0.81-1.74) | 0.371 |  |
| ≥ 111.1 | 1.49 (1.02-2.17) | 0.041 |  |
| Trp, μmol/L |  |  |  |
| < 41.6 | 1.00 |  | 0.307 |
| 41.6-53.4 | 1.21 (0.83-1.76) | 0.331 |  |
| ≥ 53.4 | 1.22 (0.84-1.79) | 0.301 |  |

Abbreviations: CVD, cardiovascular diseases; OR, odds ratio; CI, confidence interval; Glu, glutamate; Trp, tryptophan.

adjusted for age, sex, duration of diabetes, current smoking, current drinking, body mass index (≤18.5 kg/m2, 18.5-24 kg/m2, 24-28 kg/m2, ≥28 kg/m2), systolic blood pressure, high-density lipoprotein cholesterol (<1mmol/L in male or <1.3mmol/L in female, ≥1mmol/L in male or ≥1.3 mmol/L in female, Lack), low-density lipoprotein cholesterol (<2.6mmol/L, ≥2.6 mmol/L, Lack), glycated hemoglobin (<7%, 7%~8%, ≥8%, lack) , triglyceride (<1.7mmol/L, ≥1.7mmol/L, Lack), antidiabetic drugs, lipid lowering drugs and antihypertensive drugs.
